# Rapid assessment of breast tumor margins using deep ultraviolet fluorescence scanning microscopy

**DOI:** 10.1117/1.JBO.25.12.126501

**Published:** 2020-11-25

**Authors:** Tongtong Lu, Julie M. Jorns, Mollie Patton, Renee Fisher, Amanda Emmrich, Todd Doehring, Taly Gilat Schmidt, Dong Hye Ye, Tina Yen, Bing Yu

**Affiliations:** a Marquette University and Medical College of Wisconsin, Department of Biomedical Engineering, Milwaukee, Wisconsin, United States; b Medical College of Wisconsin, Department of Pathology, Milwaukee, Wisconsin, United States; c Medical College of Wisconsin, Department of Surgery, Milwaukee, Wisconsin, United States; d Abemis LLC, Cleveland, Ohio, United States; e Marquette University, Department of Electrical and Computer Engineering, Milwaukee, Wisconsin, United States

**Keywords:** breast-conserving surgery, tumor margin, fluorescence imaging, ultraviolet light

## Abstract

**Significance:** Re-excision rates for women with invasive breast cancer undergoing breast conserving surgery (or lumpectomy) have decreased in the past decade but remain substantial. This is mainly due to the inability to assess the entire surface of an excised lumpectomy specimen efficiently and accurately during surgery.

**Aim:** The goal of this study was to develop a deep-ultraviolet scanning fluorescence microscope (DUV-FSM) that can be used to accurately and rapidly detect cancer cells on the surface of excised breast tissue.

**Approach:** A DUV-FSM was used to image the surfaces of 47 (31 malignant and 16 normal/benign) fresh breast tissue samples stained in propidium iodide and eosin Y solutions. A set of fluorescence images were obtained from each sample using low magnification (4×) and fully automated scanning. The images were stitched to form a color image. Three nonmedical evaluators were trained to interpret and assess the fluorescence images. Nuclear–cytoplasm ratio (N/C) was calculated and used for tissue classification.

**Results:** DUV-FSM images a breast sample with subcellular resolution at a speed of $1.0\;\min/{\mathrm{cm}}^{2}$. Fluorescence images show excellent visual contrast in color, tissue texture, cell density, and shape between invasive carcinomas and their normal counterparts. Visual interpretation of fluorescence images by nonmedical evaluators was able to distinguish invasive carcinoma from normal samples with high sensitivity (97.62%) and specificity (92.86%). Using N/C alone was able to differentiate patch-level invasive carcinoma from normal breast tissues with reasonable sensitivity (81.5%) and specificity (78.5%).

**Conclusions:** DUV-FSM achieved a good balance between imaging speed and spatial resolution with excellent contrast, which allows either visual or quantitative detection of invasive cancer cells on the surfaces of a breast surgical specimen.

## 1 Introduction

Breast cancer afflicts millions of women in the United States and worldwide, of whom about a half to two-thirds will undergo breast conserving surgery (BCS) or lumpectomy.^1^^,^^2^ The goal of BCS is to completely remove the tumor with a narrow rim/margin of normal, unaffected breast tissue while preserving as much normal tissue as possible. Although outcomes have improved over the last decade, women with positive margins (cancer cells at the surface of the surgical specimen) still have at least a twofold increased risk of cancer recurrence,^3^^–^^5^ and thus, are often recommended to undergo additional surgery to achieve clear margins. Additional surgery is associated with additional discomfort, surgical complications, worse cosmesis, and additional emotional stress, time, and financial burdens to patients and their caregivers.^6^^,^^7^ The current re-excision rate of BCS in the United States is 14% to 18% and highly variable among surgeons, ranging from 0% to 92%.^2^^,^^8^^–^^12^ One reason why women with positive margins need to undergo re-excision surgery is that definitive pathologic margin status is typically not available until several days after surgery.

Currently, there are several different methods for intraoperative margin assessment that vary markedly in performance among studies. Radiologic examination of the resected specimen with two-dimensional (2D) mammography is largely available and rapid. However, it has low sensitivity and variable accuracy compared to other techniques and does not improve re-excision rates.^13^^,^^14^ Pilot studies evaluating intraoperative specimen radiograph using digital breast tomosynthesis may be promising (sensitivity 77% to 93% and specificity 78% to 98%), but it requires further investigation.^15^^,^^16^ Frozen section analysis is an involved pathological technique that typically samples only a portion of the surgical margin, performs poorly on fatty breast tissue, and has variable false-negative rates.^17^ In addition, this technique is extremely labor- and time-intensive, costly, requires on-site or telepathology, and significantly lengthens operating room (O.R.) time (20 to 30 min).^13^ Cytologic imprint prep analysis by touch preparation or scrape preparation is less labor-intensive (about 13 min); however, this technique requires a specialized cytopathologist.^18^ A considerable number of patients undergo BCS at an ambulatory surgery center where there is no pathologist or cytopathologist available. For these reasons, intraoperative pathology options are not routinely available or adopted.^13^^,^^19^ Lastly, the only Federal Drug Administration (FDA)-approved device for margin analysis, the MarginProbe, is a pen-like probe that utilizes radiofrequency spectroscopy to analyze tumor margins. Limitations include low sensitivity (67% to 75.2%) and specificity (46.4% to 60%) and reliance on user-guided spot scanning.^20^^,^^21^

Margins of resected lumpectomy specimens have recently been assessed using a number of emerging technologies, including optical coherence tomography (OCT),^22^^–^^25^ diffuse reflectance spectroscopy (DRS),^26^^–^^28^ DRS and fluorescence spectroscopy,^29^ Raman spectroscopy,^30^ photoacoustic tomography (PAT),^31^^–^^33^ fluorescence or polarization imaging,^34^^,^^35^ spatial frequency domain imaging (SFDI) or structured illumination imaging,^36^^,^^37^ bioimpedance spectroscopy (ClearEdge),^38^ mass spectrometry (i-Knife),^39^ and light-sheet microscopy,^40^^,^^41^ among others. A detailed review of these emerging techniques can be found in three recent comprehensive reviews.^12^^,^^42^^,^^43^ While each of these studies has shown promising results, none of the emerging imaging technologies has demonstrated the capability of analyzing an entire lumpectomy specimen (six margins) with both adequate resolution and time efficiency required in a clinical setting.

Microscopy with UV surface excitation (MUSE) is a nondestructive technology that can image fresh, unfixed tissue that is stained with multiple fluorescence dyes, thus generating fluorescence images with microscopic resolution, sharpness, and contrast.^44^ Yoshitake et al.^45^ found that the histological features of breast tissue images obtained with a high incident angle water immersion illumination MUSE system have limited correspondence with those obtained with conventional hematoxylin and eosin (H&E) histology and suggested that further development is needed for breast surgical applications. Xie et al. developed a MUSE system with a fully automated three-dimensional (3D) sample translation that can image fresh tissue at a rate of $5\;\min/{\mathrm{cm}}^{2}$, and an algorithm that can create a fluorescent analog of conventional H&E images. They further demonstrated that MUSE could provide microscopic visualization of breast margin surfaces at speeds relevant for intraoperative use.^46^

In this study, we investigated the translational potential of MUSE as an intraoperative tool for margin assessment during BCS. Specifically, we aimed to determine the features of MUSE images that can be used to distinguish fresh, unprocessed malignant from normal/benign breast tissues, the accuracy of the technology, and the speed at which a breast tissue sample can be surveyed. In order to obtain both adequate accuracy and time efficiency required in a clinical setting for margin assessment, we chose a combination of deep ultraviolet (DUV) (285 nm) excitation and low magnification (4×) with slightly reduced spatial resolution (2 to 3  μm) to achieve a faster imaging speed. High speed is necessary to survey all six margins of a lumpectomy specimen (an average size of $20\;{\mathrm{cm}}^{2}$) during surgery without causing significant O.R. delays.^28^ We purposefully focused on detecting invasive cancer cells on sample surfaces based on the 2014 Society of Surgical Oncology (SSO) and American Society for Radiation Oncology (ASTRO) Guidelines that support “no tumor cells at the inked margin” are adequate for women with stage I/II invasive cancers who undergo BCS followed by whole-breast radiation.^47^ We demonstrate that: (1) a low-cost deep-ultraviolet fluorescence scanning microscope (DUV-FSM) can rapidly image fresh breast tissues with excellent contrast at a resolution sufficient to resolve cells of different tissue types; (2) visual interpretation of MUSE images can achieve excellent sensitivity and specificity in distinguishing invasive cancer from normal breast tissue; and (3) nuclear–cytoplasm ratio (N/C) may also be used for quantitative assessment to differentiate cancer from normal breast tissue. These translational potentials of MUSE technology have not been explored in previous studies.

## 2 Methods

### 2.1 Imaging System

**Fig. 1:**
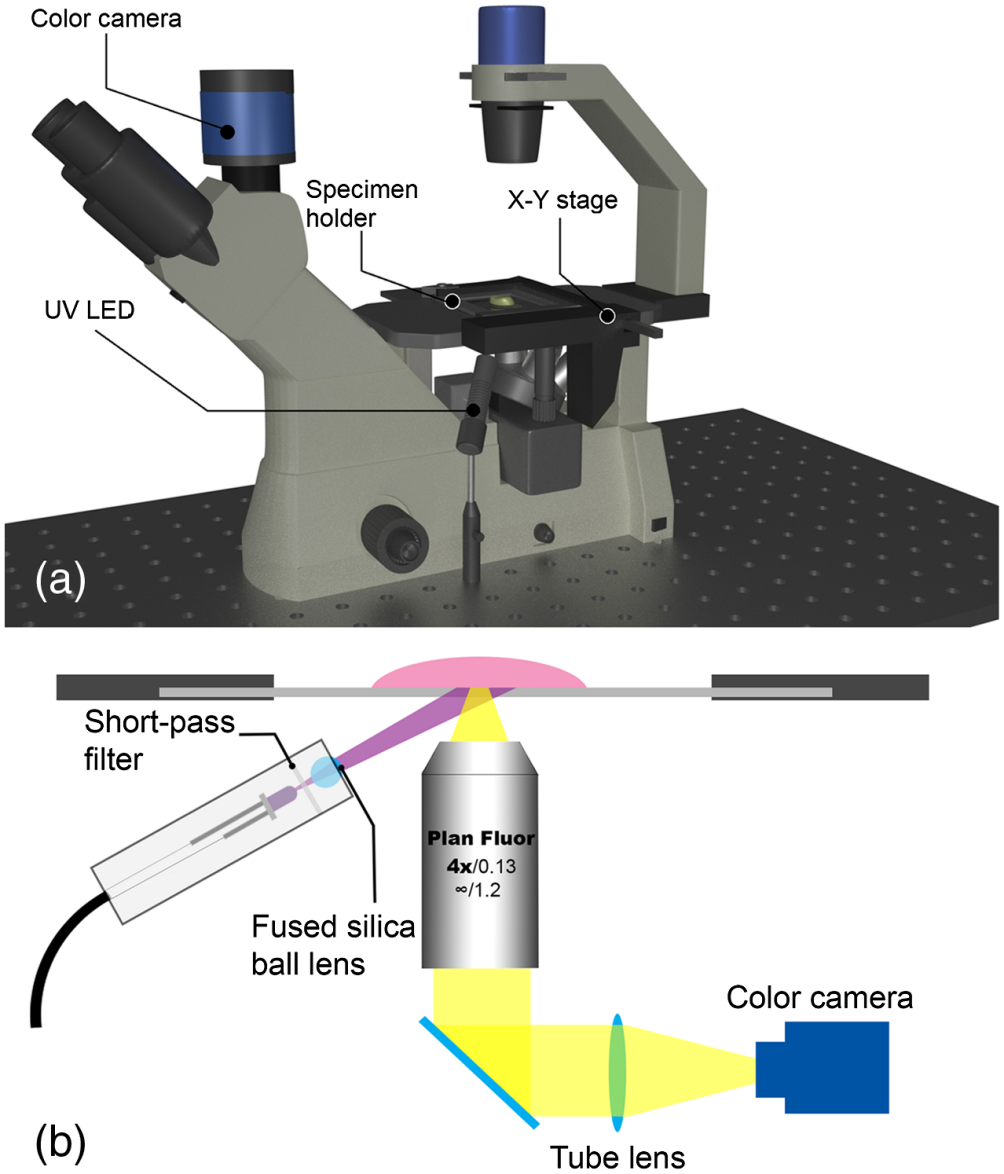
The DUV-FSM margin imaging system: (a) principle schematic of the system and (b) the simplified imaging light path. A 45-mW, 285-nm LED with a short-pass filter and a fused silica ball lens has been added to the inverted fluorescence microscope for fluorescence excitation. Fluorescence emission is collected by a Plan Fluor 4× (numerical aperture=0.13) objective. The excitation/emission filter block of the microscope is switched to the empty position. Large specimen mosaic scanning is achieved by a motorized XY stage.

We have converted an inverted microscope to a DUV-FSM to image the surfaces of fresh tissues from breast surgical specimens. A schematic of the DUV-FSM system is shown in Fig. 1. A 285--nm LED (M285L4, Thorlabs, Newton, New Jersey) is mounted on the right side of an inverted fluorescence microscope (EXI-310, Accu-scope, Commack, New York) for oblique back-illumination. A 325-nm short-pass filter (XUV0325, Asahi Spectra, Torrance, California) is placed in front of the LED to block emission spectrum tails in the visible range, avoiding possible overlap with fluorescence signals. A fused silica ball lens (model #67-388, Edmund Optics, Barrington, New Jersey) is used as a condenser to converge the LED radiation for a smaller illumination field and improved power density. The LED, short pass filter, and ball lens are mounted inside a lens tube. A 3D-printed arm holds the lens tube and is mounted on an optical post to allow for easy adjustment of the LED height and illumination angle so that the illumination area is slightly larger than the field-of-view (FOV) of the microscope objective. The power density of the LED light at the tissue surface was measured at 9.4 mW/cm^2^. Once the position of the LED was optimized, the entire system was fixed on an optical breadboard. To image a lumpectomy specimen, the specimen is placed on one of its six margins in a 70-mm-diameter quartz dish (1.0-mm thick) to minimize autofluorescence of the glass. The quartz dish is mounted on a robotic, stepper-motor controlled XY stage custom designed for fast mosaic imaging (ABĒMIS LLC, Cleveland, Ohio). The excitation/emission filter block of the microscope is switched to the empty position so that the fluorescent signals of multiple fluorophores can be captured by a color camera without having to switch emission filters during the imaging process. A cooled, USB3.0 camera (MTR3CCD06000KPA, Hangzhou ToupTek Photonics Co., Ltd, Hangzhou, China) was selected for its large image sensor and pixel size, very low dark noise, and high image transfer speed, which are very important for fast image acquisition in intraoperative margin assessment. The camera has 2748×2200  pixels, a pixel size of 4.54  μm, and active area of $14.6\times 12.8\;{\mathrm{mm}}^{2}$. A 4× apochromatic long working distance objective lens with a numerical aperture of 0.13 was selected as a compromise between good lateral resolution (2 to 3  μm) and a large effective imaging area of $3.48\times 2.78\;{\mathrm{mm}}^{2}$. The FOV of the objective lens is slightly larger than the imaging area of the camera to avoid distortions at the edge of the FOV. The microscope is housed inside a dark enclosure to prevent personnel exposure to DUV light and to eliminate background from room light.

### 2.2 Breast Tissue Sample Preparation

Forty-seven fresh human breast tissues obtained from lumpectomy and mastectomy specimens were acquired from the Medical College of Wisconsin (MCW) Tissue Bank. Information about the tissue subtype, number of samples, and surface area are provided in Table 1. Specimens were grossly examined and procured by Tissue Bank staff (MP) then placed in phosphate-buffered saline (PBS) solution, transported on ice to the research lab immediately, and stored in a refrigerator. Samples were imaged on the day of excision. Propidium iodide (PI, P21493, Thermo Fisher Scientific, Waltham, Massachusetts) was used for nuclear staining and eosin Y (EY, 230251-25G, Sigma-Aldrich, St. Louis, Missouri) for staining of cytoplasm and connective tissues. Both PI and EY can be effectively excited at 285 nm. PI has a fluorescence emission in the yellow-red spectral range and EY has an emission in the green-yellow spectral range. For staining, PI and EY were dissolved in PBS (pH 7.2) to a concentration of 100  μg/ml and 1.0  mg/ml, respectively. Each specimen was stained in PI solution for 1 min, then in EY solution for 20 s, and finally rinsed in PBS for 10 s. Once staining was completed, the specimen was placed onto the quartz plate of the specimen holder. A wide pallet knife was used to gently flatten the tissue against the quartz plate to remove air bubbles between the tissue and plate. Once the tissue was in the correct position, excess liquid was removed from the edges using a Kimwipe. Although both sides of the samples were imaged, the samples were thin and the two surfaces were similar. Thus, only images from one side were included in data analysis. All handling and safety guidelines for biohazards were carefully followed. The study was exempt from the Institutional Review Board and Institutional Biosafety Committee reviews.

**Table 1:** Tissue types, number of samples, surface area, and number of patches ($2\times 2\;{\mathrm{mm}}^{2}$).

Tissue type	No. of samples	Surface area (${\mathrm{cm}}^{2}$) (median)	No. of patches/sample (median)	Total no. of patches
Invasive ductal carcinoma (IDC)	25	0.45 to 5.5 (1.2)	3 to 48 (15)	445
Invasive lobular carcinoma (ILC)	6	0.91 to 3.9 (2.35)	4 to 55 (15.5)	133
Adipose-rich normal	3	3.9 to 5.29 (4.84)	48 to 70 (58)	176
Fibrous/glandular-rich normal	13	2.4–9 (4.41)	36–141 (91)	1135

### 2.3 Imaging Protocol

The specimen holder loaded with a tissue specimen was immobilized on the motorized XY stage after a specimen size measurement by a caliper. The focal plane was set at the bottom surface of the specimen. Mosaic images were collected with conservative overlapping regions of 0.75 mm in the X direction and 0.60 mm in the Y direction for a tradeoff between speed and stitching accuracy. The number of scanning steps is decided by the specimen size, effective imaging area, and the dimension of overlapping. The temperature of the camera was set to −18°C to reduce the electronic noise level. All images of each specimen were captured with a constant exposure time ranging from 50 to 100 ms, depending on the sample tissue type. The image acquisition and motor movements were controlled by a customized software developed in Microsoft Visual C#.NET. Image files were saved in TIFF format with 2748×2200  pixels. After imaging, the raw images were transformed to hue–saturation–lightness (HSV) color space images. The open source image processing package Fiji^48^ was used to process the tissue images. A Fiji plugin named BaSiC^49^ was applied to the saturation and lightness channels to correct for background and shadings caused by uneven and tilted illumination of a single DUV LED. The color space transform is necessary to preserve the original color information during illumination correction. After transforming back to red-green-blue (RGB) color space, image stitching was performed using a Fiji plugin developed by Preibisch et al.^50^ Finally, histogram equalization was applied to the R and G color channels of the stitched image to enhance the visual contrast.

### 2.4 Histopathology Evaluation

Routine histopathology was used for final diagnosis of tissue samples. Fereidouni et al.^44^ previously shown that PI and EY staining does not interfere with subsequent histopathology processes. Following DUV-FSM imaging, tissue specimens were returned to MCW Tissue Bank for formalin-fixed paraffin-embedded (FFPE) tissue processing. In order to obtain full face sections for histologic evaluation, an average cut depth of ∼200  μm into the embedded tissue block was used during microtomy. Tissue sections were transferred to glass slides and stained with H&E. All slides were digitalized by a Panoramic 250 Flash II slide scanner (3DHistech Ltd., Budapest, Hungary). An unblinded qualitative side by side comparison of H&E and MUSE images was performed by an experienced breast pathologist (JMJ).

### 2.5 Visual Inspection of MUSE Images

Visual inspection of MUSE images was performed by three nonmedical evaluators to assess the accuracy of nonpathologists to differentiate cancer from noncancer tissue. The 47 breast tissue samples were divided into two groups: training and test groups. The training group included three invasive carcinomas (two IDC and one ILC) and two normal tissues (one fibrotic and one adipose-rich breast sample), while the test set included 5 ILC, 23 IDC, 2 adipose-rich, and 12 fibrotic/nonadipose-rich normal samples. Three nonmedical evaluators (TGS, DHY, and AE) who were blinded to the pathological diagnosis were trained by the pathologist (JMJ) and imaging engineer (TL) during a 1-h session to visually identify the diagnostically useful features (such as adipose, ducts, cell density, infiltration, etc.) in the training MUSE images using the associated H&E images. After training, each evaluator was provided MUSE images of samples in the test group without access to correlative H&E images. The evaluators interpreted MUSE images and provided a diagnosis (invasive carcinoma versus normal) for each of the test samples.

### 2.6 Quantitative Image Analysis

Quantitative analysis was applied to DUV tissue images to extract diagnostically useful parameters that may be useful for detecting positive tumor margins of lumpectomy specimens during BCS. Previous studies have shown that breast cancer cells have irregular cell size and shape, enlarged nuclei, and increased N/C.^51^^,^^52^ In this study, we investigated the feasibility of using N/C as a biomarker to differentiate invasive carcinoma from normal breast parenchyma at the surface of the tissue samples. Tumor region(s) on the stitched MUSE image was outlined based on the corresponding H&E image. Since PI-stained cell nuclei primarily emit lights in the yellow-red wavelength range, only the red channel (R-channel) of the stitched images in RGB color space was extracted and used to calculate the N/C.

**Fig. 2:**
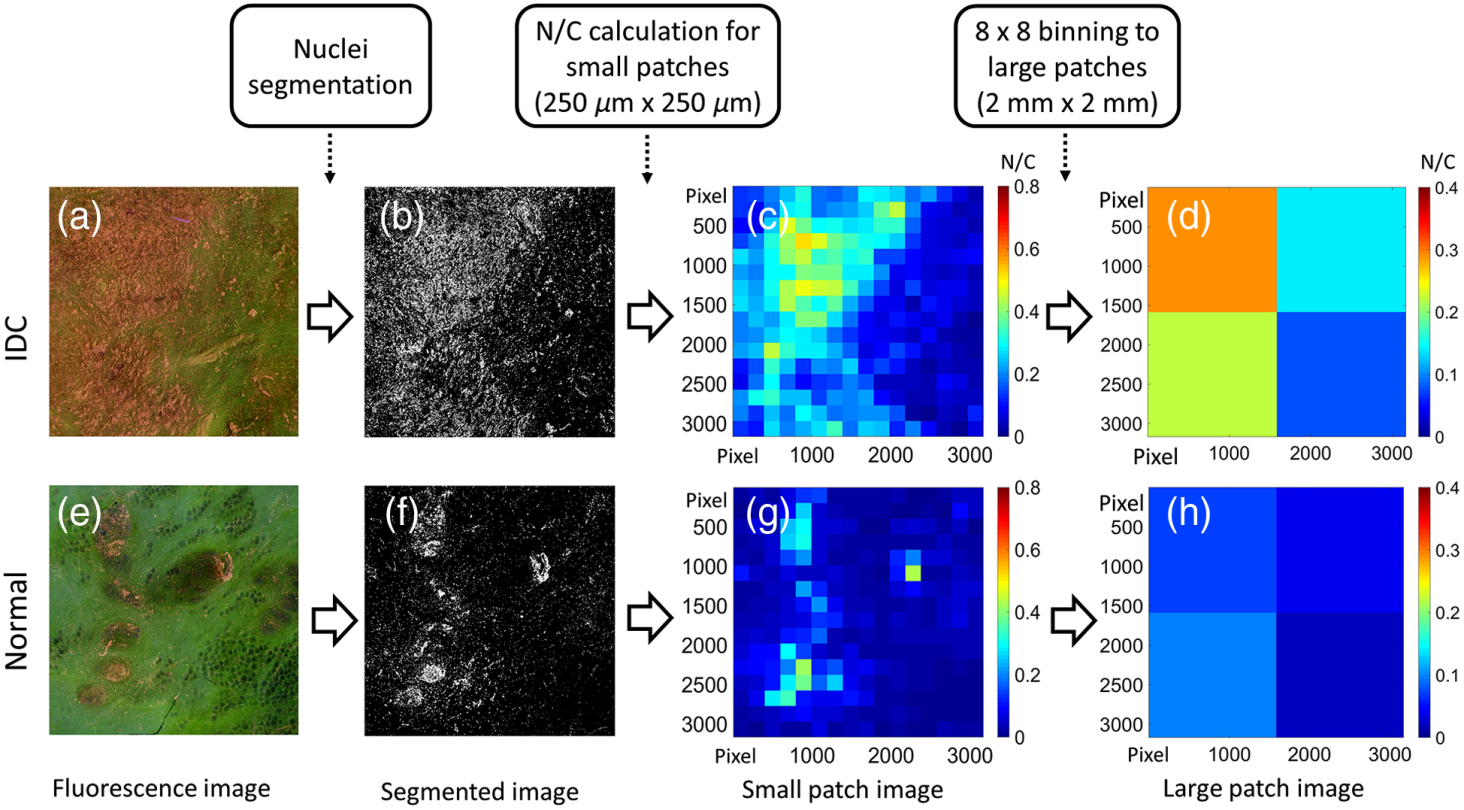
Demonstration of N/C calculation process with (a)–(d) an IDC and (e)–(h) a normal sample. (a) and (e) A $4\times 4\;{\mathrm{mm}}^{2}$ area from the fluorescence images of an IDC and a normal sample, respectively. (b) and (f) The binary image after nuclei segmentation from the same region as in (a and e). (c) and (g) The calculated N/C map of small patches ($250\times 250\;{\mu m}^{2}$), with color bar on right signifying N/C values. (d) and (h) N/C of large patches ($2\times 2\;{\mathrm{mm}}^{2}$) obtained by averaging over 8×8 small patches from (c) and (g). As seen in color bars on right of N/C maps, low N/C ratios are represented by blue shades; higher N/C ratios are depicted by green, yellow, orange and red shades.

The process for N/C calculation is shown in Fig. 2. First, the R-channel image (not shown) was extracted from the color MUSE image (a) and (e). Segmentation of the R-channel image was implemented by combining edge detection and intensity thresholding.^53^ The edge detection Sobel operator with adaptive threshold detects the edge information, while intensity thresholding eliminates textures caused by other features. The intensity threshold was set to 70% of the 1% brightest pixels in the image, based on our experience. The segmented binary images (b) and (f) were divided into a set of small patches of $250\times 250\;{\mu m}^{2}$ (or 198×198  pixels) in size, and N/C was calculated for each small patchs (c) and (g) by dividing the number of white pixels by the total number of pixels within the patch. Then, N/Cs of neighboring 8×8 small patches were averaged and merged to form a large patch of $2\times 2\;{\mathrm{mm}}^{2}$ in size, which resulted in an N/C ratio map with 2×2 large patches (d) and (h). A window size of $2\times 2\;{\mathrm{mm}}^{2}$ was selected to match the spatial resolution of standard breast pathology, which samples at a step of 2 mm. Larger patches were used to reduce the sensitivity to small features with high cell density, such as lobules, ducts, and blood vessels. In general, larger patch size results in lower spatial resolution and sensitivity for cancer cell detection, but also a lower false positive rate. To minimize the effect of tissue patches at the boundaries, large patches with more than half (or 32) small patches that have no cells (i.e., N/C=0) were excluded in further analysis.

The large patches were manually classified into adipose-rich and nonadipose-rich normal, ILC and IDC in accordance with H&E images. The number of large patches per tissue sample and total number of patches for each tissue subtype are presented in Table 1. This resulted in a total of 1889 large patches of N/C images, including 445 patches from 25 IDC, 133 patches from 6 ILC, 176 patches from 3 adipose-rich, and 1135 patches from 13 nonadipose-rich normal tissues. All patches were used for the following comparison and classification studies. Mean N/C was compared between the four tissue subtypes (IDC, ILC, adipose-rich, and nonadipose-rich normal) and between invasive (IDC, ILC) and normal using generalized estimating equations (GEE) to account for repeated observations per sample. Tukey’s adjustment was used for multiple pairwise comparisons.^54^ For classifications, receiver operating characteristic (ROC) curves were constructed using patch-level N/C to predict invasive versus normal tissue, IDC versus ILC among invasive samples, and adipose-rich versus nonadipose-rich tissue among normal samples. The Youden Index, which weighs false positive and false negative errors equally, was used to determine the cutoff point for the calculation of patch-level sensitivity and specificity in differentiating invasive and normal tissue. The analysis was done using SAS 9.4 (SAS Institute, Cary, North Carolina).

## 3 Results

### 3.1 Breast Tissue Images

**Fig. 3:**
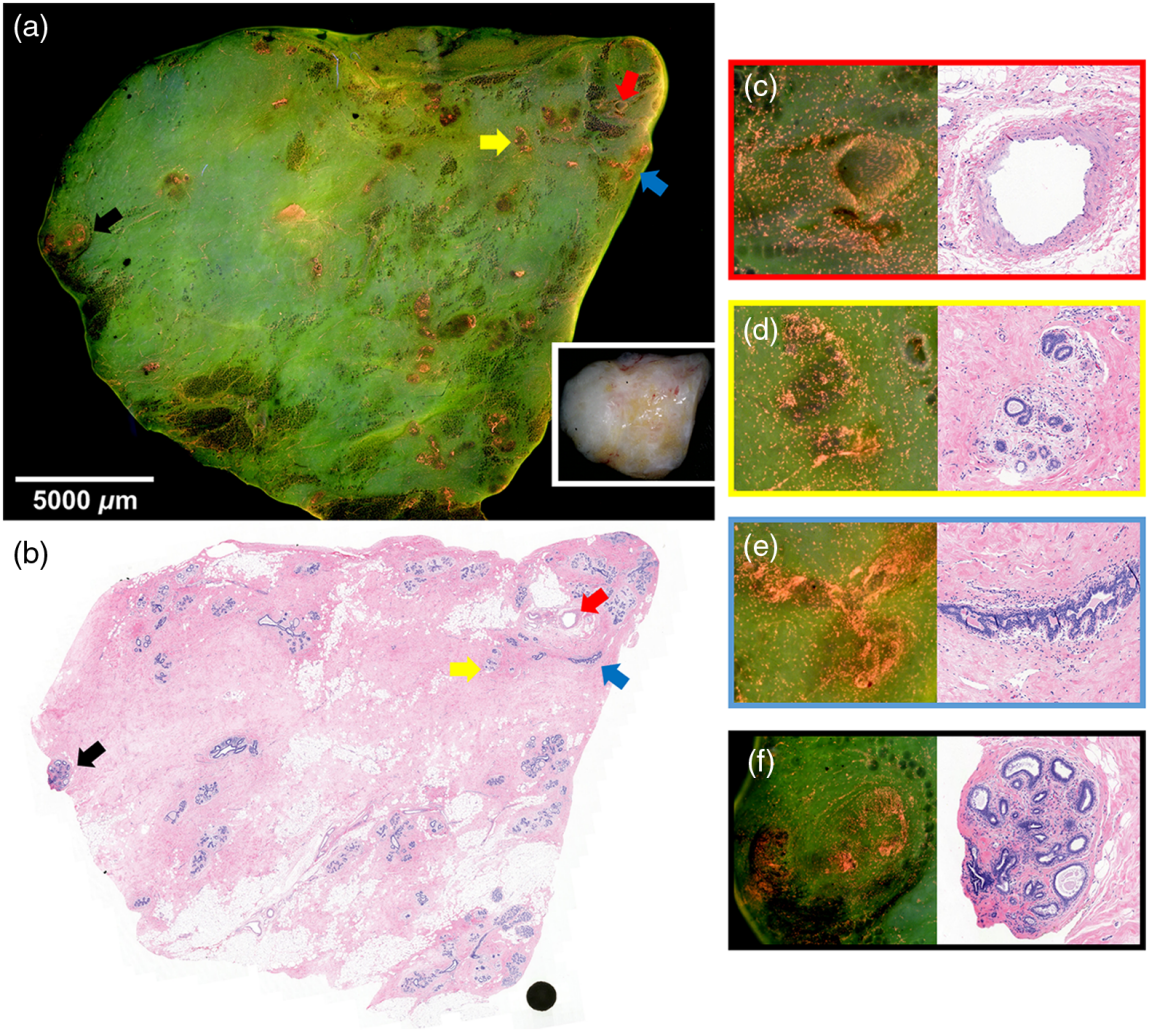
Normal human breast tissue. (a) The fluorescence image of a nonadipose-rich sample ($21\times 21\;{\mathrm{mm}}^{2}$) of benign breast glands with loose connective tissues in light green and interspersed fibroadipose tissue stroma in dark green, with the specimen photo in the white box and (b) corresponding FFPE H&E image. Zoomed fluorescence and H&E images of: (c) muscular-walled blood vessel is indicated by the red arrow, (d) small lobule indicated by the yellow arrow, (e) duct indicated by the blue arrow, and (f) lobule with mild adenosis and dilated glands indicated by the black arrow.

The study resulted in stitched MUSE images and FFPE H&E images obtained from 47 breast specimens. The average imaging speed was $1.0\;{\min/\mathrm{cm}}^{2}$. DUV fluorescence and H&E images of one sample from each tissue type are presented below. Figures 3(a) and 3(b) show fluorescence and H&E images of a nonadipose-rich sample of benign breast glands with interspersed fibroadipose tissue stroma. Most of the sample is loose connective breast tissue with low nuclear density and appears light green. Tubular-shaped structures, e.g., blood vessels [Fig. 3(c)], ducts [Fig. 3(e)], and lobules [Figs. 3(d) and 3(f)], are easily identifiable. The two-layers of epithelia of the ductal-lobular system have higher nuclear densities than blood vessels. Terminal duct lobular units exhibit high nuclear density and their shapes are more complicated than ducts and blood vessels. In addition, lobules have clustered foci of dense nuclei comprising glands while ducts tend to have a solitary structure.

**Fig. 4:**
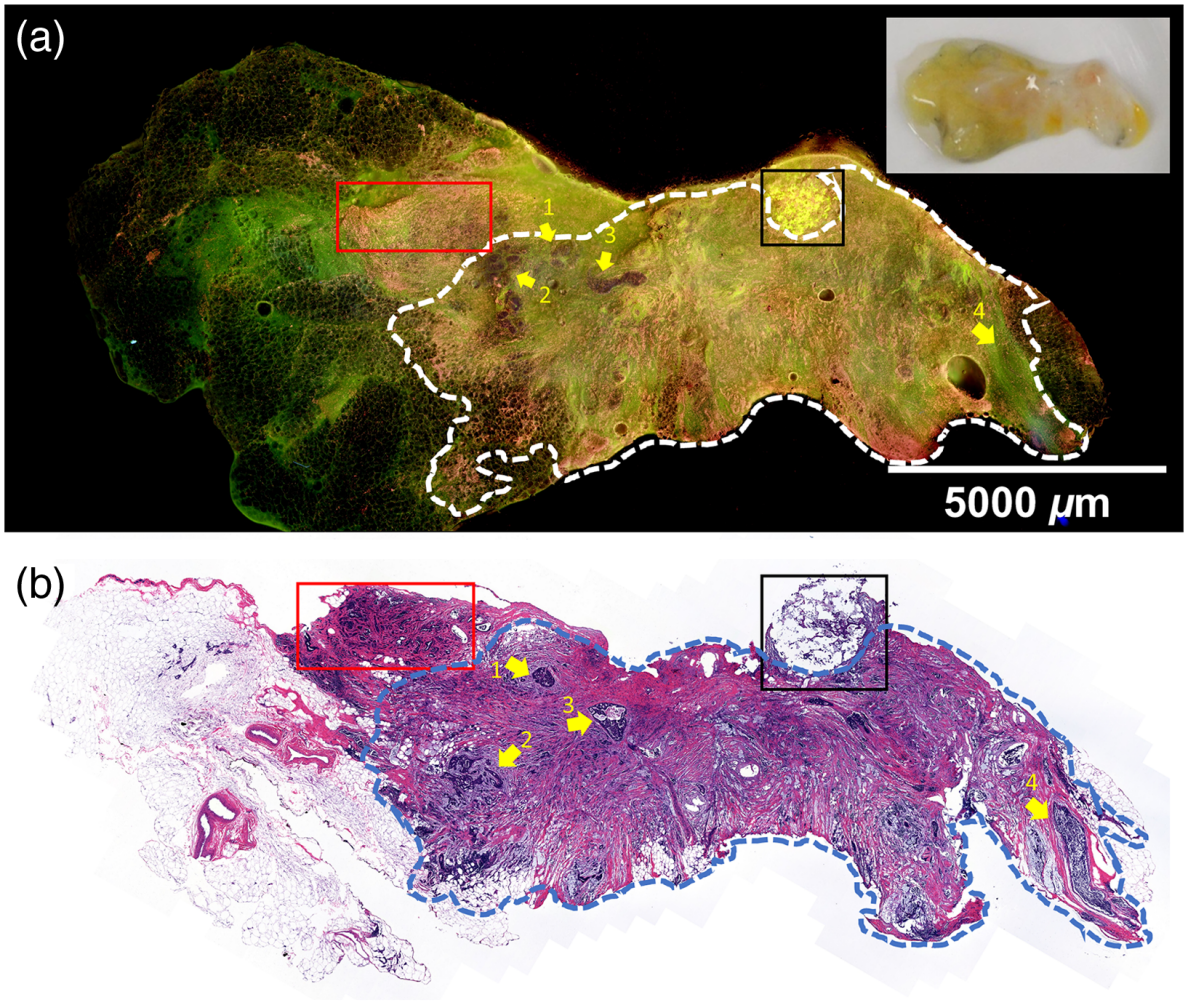
Human breast tissue with ILC and DCIS. (a) The fluorescence image of an ILC sample ($20\times 10\;{\mathrm{mm}}^{2}$) with adjacent adipose tissue at the two ends. A specimen photo is included in the upper right corner. (b) FFPE H&E image of the same sample. ILC cells enclosed by the dashed lines appear pink and yellow in the DUV fluorescence image and can be easily distinguished from the adipocytes at the left and the right sides, which appear as individual or clusters of rounded small droplets in dark green with hypo-scattered bright pink nuclei. DCIS sites are pointed by arrows 1, 2, 3, respectively. The DCIS indicated by arrow 4 is not visible in the fluorescence image, likely because it is slightly below the surface. Biopsy site is highlighted by the black box. A biopsy site usually forecasts abnormalities within the site or nearby. Benign adenosis is highlighted by the red box.

Fluorescence and H&E images of a grade one ILC with multiple foci of ductal carcinoma *in situ* (DCIS) and surrounding adipose tissue are shown in Fig. 4. ILC cells in the middle (enclosed by the dashed lines) appear in pink and yellow in the DUV fluorescence image [Fig. 4(a)] and can be easily distinguished from the adipocytes at the left and the right sides, which appear as individual or clusters of rounded small droplets in dark green with hyposcattered bright pink nuclei. Tumor regions exhibit infiltrating single cells characteristic of ILC in both the H&E and MUSE images. Four areas of DCIS were identified in the H&E image [Fig. 4(b)], three of which (indicated by yellow arrows 1 to 3) were on the surface and appeared to be dark or have loss of fluorescence. The shallow penetration depth (∼20  μm^45^) of DUV lights makes DCIS below the surface invisible, such as the one indicated by arrow 4 in the H&E image. A biopsy site (black box) shows higher brightness in yellow with lighter background color. The benign adenosis region in the red box also has a high nuclear density but lacks single file infiltrating patterns seen in tumor regions. Air bubbles also appeared in the fluorescence image, but careful specimen handling and flattening can alleviate or avoid such artifacts.

**Fig. 5:**
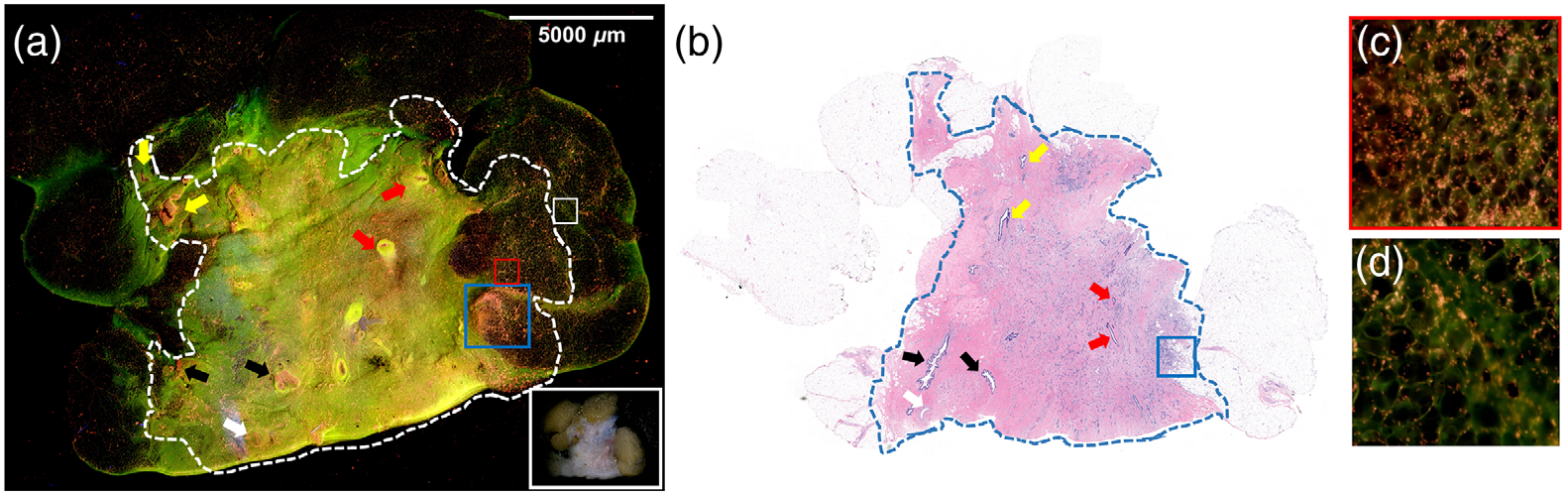
Human breast tissues with IDC. (a) The fluorescence image of an IDC sample ($25\times 18\;{\mathrm{mm}}^{2}$) with three sides surrounded by adipose tissues. The specimen photo is presented in the white box. (b) The FFPE H&E image of the same sample. The tumor region is enclosed within the dashed lines. Benign ducts are indicated by red, yellow, and black arrows from different sites. White arrow indicates the blood vessel. High nuclear density at the interface between fibrotic stroma and adipose tissue is highlighted by the blue box. The areas highlighted by the red and white boxes are shown in (c) and (d), respectively.

Fluorescence and H&E images of a grade 2 IDC surrounded by adipose tissue are shown in Figs. 5(a) and 5(b). Adipocytes on the left, right, and upper sides appear darker in green than the epithelial and connective tissues. Like the ILC specimen above, IDC cells inside the dashed line appear in pink and yellow and can be easily distinguished from the adipocytes in dark green and epithelial and connective tissues in light green in Fig. 5(a). Of note, the right side of the tumor, such as the area highlighted by the red box, shows tumor infiltration into the neighboring adipose tissue, better visualized at higher power, as shown in Fig. 5(c). Particularly, the tumor area highlighted by the blue box has very high nuclear density and is at the interface between fibrotic stroma and adipose tissue, which correlates well with the corresponding H&E image. In comparison, the area on the right side of the dashed line, representing normal adipose tissue, as highlighted in the white box shown in Fig. 5(d), has much lower nuclear density. A portion of the upper side of the specimen was compressed during imaging and shows clear folds. A thorough check before imaging can avoid this issue.

**Fig. 6:**
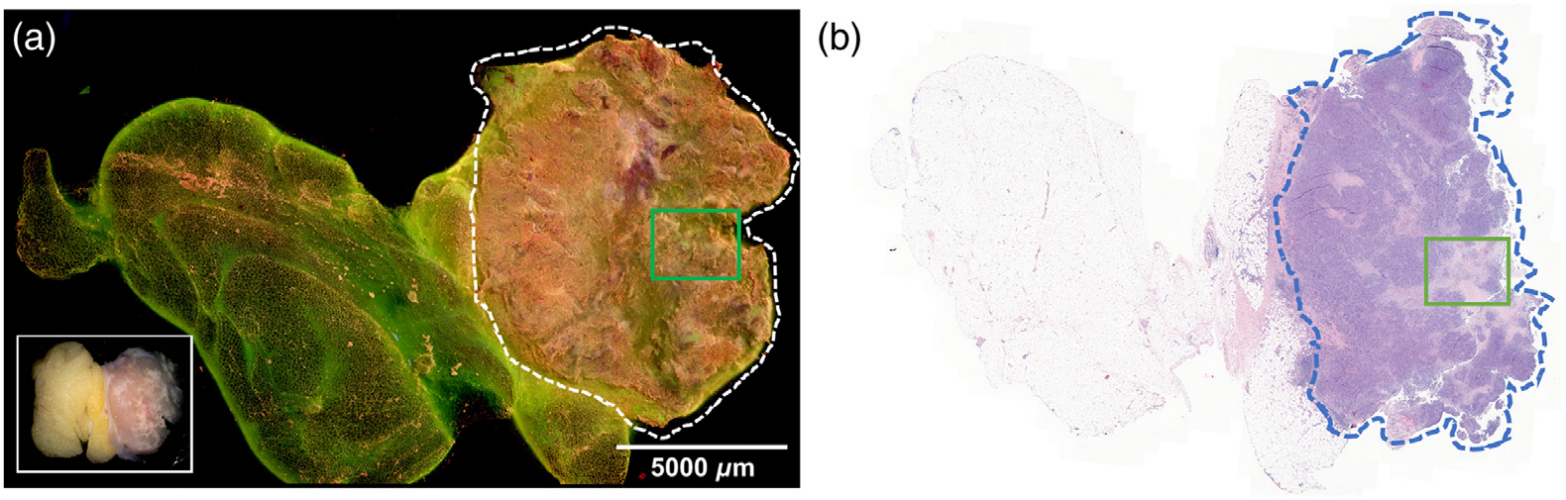
IDC sample with necrosis. (a) The fluorescence image of a high-grade IDC sample ($25\times 17\;{\mathrm{mm}}^{2}$) with necrosis and the specimen photo in the white box. (b) FFPE H&E image of the same sample. The tumor region is enclosed within the dashed lines and the region in the green box exhibits necrosis.

The fluorescence and H&E images of a high-grade IDC specimen with necrosis and surrounding adipose tissue are shown in Figs. 6(a) and 6(b), respectively. Again, adipose tissue on the left side appears dark green and the tumor region inside the dashed line has high nuclear density and poorly differentiated cells. Foci of necrosis are observed from the fluorescence image and corresponding positions on the H&E images which show weaker (pink) nuclear staining and loss of cellular detail. The upper left corner of the MUSE image in Fig. 6(a) includes some pink areas with high cell density, but no cancer cells are identified in the corresponding H&E image in Fig. 6(b). Because the H&E image was obtained from a tissue slide below the sample surface, there is not enough information to determine whether there were some cancer cells on the surface of the specimen used for fluorescence images.

### 3.2 Performance of Visual Interpretation of MUSE Images

Results from visual interpretation of the MUSE images of the 42 tissue samples in the test group by the three evaluators are summarized in Table 2. Evaluator A identified all cancer samples correctly, while evaluators B and C each missed one (false negative rates of 3.6%). Evaluators A and evaluator B identified all normal samples correctly, but evaluators C interpreted three normal samples as cancer. Overall, the three evaluators achieved an average sensitivity and specificity of 97.62% and 92.86%, respectively, and an accuracy of 96.03%. Evaluators spent ∼1 to 2 min to review the MUSE image of a test sample before making a diagnosis.

**Table 2:** Results of visual diagnosis of the 42 breast samples in the test group.

	Evaluator A		Evaluator B		Evaluator C		Average
	Cancer	Normal	Cancer	Normal	Cancer	Normal	
Path cancer	28	0	27	1	27	1	
Path normal	0	14	0	14	3	11	
Sensitivity	100%		96.43%		96.43%		97.62%
Specificity	100%		100%		78.57%		92.86%
Accuracy	100%		97.62%		90.48%		96.03%

### 3.3 Quantitative Analysis by N/C Values

**Fig. 7:**
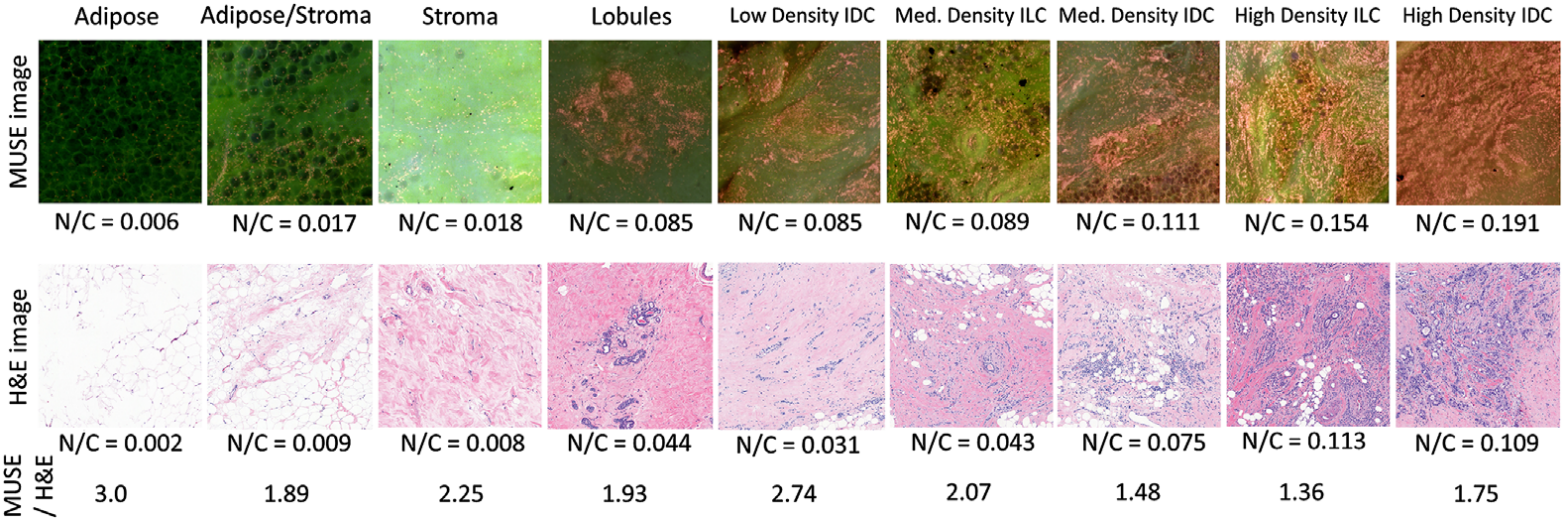
Comparison of N/C values extracted from MUSE and H&E images of representative tissue types.

The 1889 large patches of N/C images from the 47 breast tissue samples were manually classified into adipose-rich, nonadipose-rich normal, ILC and IDC tissues in accordance with the H&E images. Figure 7 shows the calculated N/C values from the MUSE and H&E images for seven representative tissue samples. The N/C values extracted from the MUSE images are all higher (MUSE/H&E=1.36∼3.0) than those extracted from the H&E images, which may be attributed to the larger sectioning depth (∼20  μm) of the DUV light than the thickness of a paraffin section (4 to 5  μm) and the lower magnification (4×) used by the DUV-FSM system compare to the 40× magnification used for routine H&E images. In general, the higher the cell density of the tissue sample, the lower the MUSE/H&E ratio. Most importantly, the MUSE images retain the contrast between invasive and normal/benign breast samples, and in both methods, lobular tissues are harder to separate from low and medium density IDC and ILC samples.

**Fig. 8:**
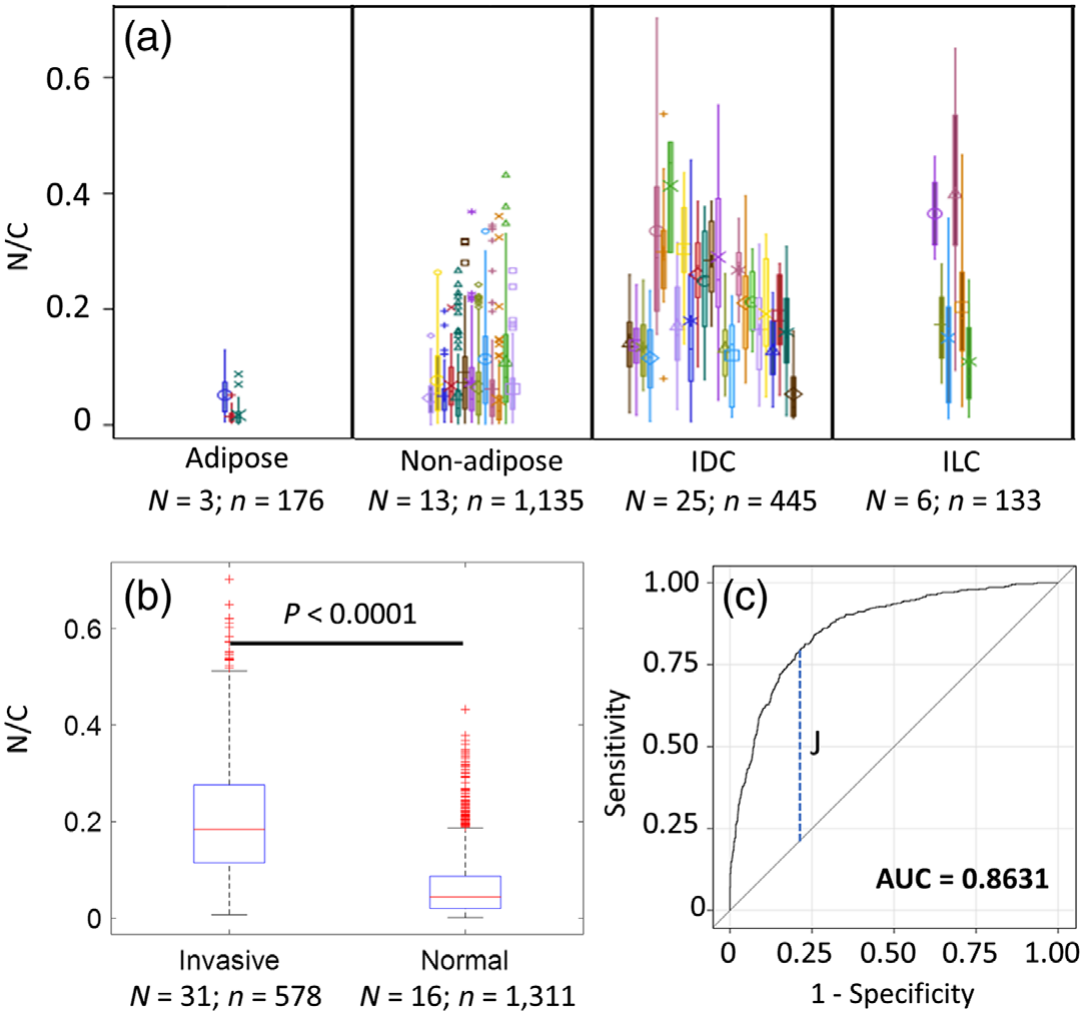
Results of statistical comparison and classification. (a) The boxplot of N/C by specimen subtype (adipose-rich, nonadipose-rich normal, IDC, and ILC). Each color in each tissue type includes all patches from that sample. N represents the number of samples and n is the total number of patches from those samples. (b) The boxplot of two-class classification. Outliers are represented by red crosses in (b). (c) ROC curve for classifying patch-level normal and invasive tissue. J is the Youden’s index.

Boxplot of N/C per sample of the four tissue types is presented in Fig. 8(a). The calculated mean patch-level N/C value was 0.0280±0.0098 for adipose-rich (n=176), 0.0706±0.0059 for nonadipose-rich normal tissues (n=1135), 0.2013±0.0150 for IDC (n=445), and 0.2307±0.0456 for ILC (n=133). The GEE model determined that there was a significant difference (p<0.001) among the four tissue subtypes except between ILC and IDC (p=0.54). Figure 8(b) shows boxplots of two-class comparison, showing N/C of invasive carcinoma (IDC and ILC) to be significantly different (n<0.0001) from normal tissues. In the ROC curve in Fig. 8(c), the area under the curve was 0.8631 and, when N/C=0.093982 was used as threshold, the patch-level sensitivity, and specificity in differentiating invasive carcinoma from normal tissues were 81.5% and 78.5%, respectively.

## 4 Discussion

This study investigated the feasibility of using DUV fluorescence scanning microscopy as a potential intraoperative tool to detect positive margins of freshly excised breast tumor specimen during BCS. Propidium iodide and eosin Y have been used to stain the tissues for enhanced fluorescence contrast between invasive and normal/benign breast tissues. Our MUSE images show excellent visual contrast in color, tissue texture, cell density, and shape between ILC, IDC, and DCIS and normal tissues. Nonexpert evaluators with minimal training were able to distinguish IDC and ILC accurately and quickly from normal breast tissues by simple visual interpretation of MUSE images. As the majority of breast cancer surgery is performed in settings where a pathologist is generally not readily available, we chose nonmedical staff to evaluate the MUSE images to demonstrate that visual evaluation of the MUSE images is simple, fast, and accurate, and does not require significant training or expertise. Therefore, the DUV-FSM should be practical and generalizable to most settings as it does not require specialized resources or training. In addition to providing surgeons high contrast images for visual inspection, N/C from MUSE images showed significant difference [Fig. 8(b)] between invasive carcinoma and normal breast tissues. Reasonable patch-level sensitivity and specificity in differentiating invasive carcinoma from normal was achieved using a trained statistical classification model and N/C as a single discriminator. N/C has not been extensively used as a parameter for intraoperative assessment of breast margins.

DUV-FSM has several important features. First, DUV-FSM can image large margins of variable size with subcellular resolution. Since the size of lumpectomy margins varies and most positive margins typically involve small microscopic foci of cancer,^55^ a device with both large margin coverage and microscopic resolution is highly desirable to rapidly evaluate lumpectomy margin status intraoperatively with both low false-negative and false-positive rates. However, many technologies currently under investigation are either a point device (e.g., optical spectroscopy, i-Knife, and MarginProbe) or a high-resolution device with very small field of view (e.g., OCT and confocal) that requires excessive time to manually scan a surgical margin or a wide-field imaging device with very low spatial resolution (e.g., fluorescence imaging and SFDI). Compared to near-infrared light and visible light, using DUV light to detect tumor at the surface should result in subcellular spatial resolution (2 to 3  μm) even with a low magnification objective (e.g., 4×), which is sufficient in resolving cellular structures and cell nuclei in *ex vivo* breast tissue, and much lower background noise caused by fluorescence emission from deeper cells and tissue scattering. With automated scanning and stitching, lumpectomy margins of different size can be easily surveyed by varying the number of 4× images in the X and Y directions in the user interface of the DUV-FSM software. Hence, the DUV-FSM has a high imaging speed at a spatial resolution that is adequate to achieve a high accuracy for detection of a small amount of cancer cells. This feature is clinically critical because it allows tumor margins of a wide range dimensions to be imaged rapidly during BCS.

The DUV-FSM system described here, like other MUSE devices,^44^^–^^46^ directly images fresh specimens without the need for complex intraoperative radiography and/or pathology processing. This method is extremely simple, easy to use, relatively low in cost, and does not require radiology, pathology, or cytology expertise, thereby making it attractive to community hospitals and surgery centers where most BCS procedures are performed. Using DUV-FSM for intraoperative margin imaging is nondestructive to the specimen and does not negatively impact postoperative pathology, which remains the gold standard for margin assessment. The sensitivity and specificity for rapid visual inspection of MUSE images were high, even among nonexpert evaluators with minimal training, and thus should not pose significant barriers to surgeon adoption or operating room workflow as most surgeons already interpret specimen radiograph results. To our knowledge, this study is the first to report the sensitivity and specificity of MUSE technology for detection of cancer versus noncancerous breast tissue.

Previous MUSE studies focused on creating H&E mimicking images by color mapping for histopathological assessment, which requires a pathologist to interpret the images. While creating H&E mimicking images from MUSE images is necessary to understand if MUSE can provide the same or equivalent diagnostically useful information as one can get from routine pathological analysis, it is also important to investigate how this technology may be used in the intraoperative clinical setting assessing lumpectomy margin status where large and variable margin coverage, high speed, and simplicity are key factors. This DUV-FSM study emphasizes the importance of providing surgeons diagnostically interpretable information about margin status within minutes. We aim to generate high contrast images that allow a surgeon to visually diagnose or a computer algorithm (e.g., statistical or machine learning model) to determine if a margin is positive and re-excision is necessary to achieve a negative margin during the primary surgery. Equally important is determining if margins are negative to prevent the unnecessary removal of additional breast tissue. Best efforts are focused on achieving a balance between high resolution to obtain more cellular and subcellular details about the tissue surface and fast scan speed that allows specimen processing, imaging, and interpretation of all six margins of a lumpectomy specimen in the O.R. Speed is critical for intraoperative margin assessment as every minute in the operating room costs around at least $36.^56^ The tradeoff between resolution and speed is necessary until a practical margin tool that can achieve both high resolution and high speed is developed. Using a low 4× magnification objective with an FOV over 4 mm in diameter, simultaneous excitation of two fluorescence dyes with distinct emission color (PI and EY), and a universal serial bus (USB) 3.0 color camera to detect the fluorescence signals significantly reduces the scan time for a large specimen to $1\;{\mathrm{cm}}^{2}/\min$. The DUV-FSM method requires 80 s for sample staining and ∼1  min for image processing. In contrast, the Xie system requires 5 min for staining, 5 min to scan a $1.0\;{\mathrm{cm}}^{2}$ margin, and 5 min for image processing to create fluorescent analog of H&E staining.^46^

This study has some limitations. First, this study purposefully focused on invasive cancer so pure DCIS samples were not included in the study. We are aware that SSO/ASTRO released consensus guidelines on margins in 2016 for BCS with whole breast radiation in the setting of DCIS (with or without microinvasive component), which accounts for about 25% of all breast cancers. The recommendation is to achieve a 2-mm margin to minimize the risk of tumor recurrence in the same breast and emphasizes the importance of clinical judgment in determining selective re-excision for patients with negative margins <2  mm.^57^ We acknowledge that DUV-FSM, as well as other MUSE devices, will not be able to assess margins to 2 mm and close margin status (tumor cells present within 2 mm of the surface but not at the surface) will need to be determined on final routine pathological assessment. However, our preliminary work demonstrates the possibility of using DUV-FSM to detect positive margins for DCIS (see Fig. 4), which should translate into decreased re-excision rates in positive-margin cases. Recent data from two large studies showed that women with DCIS who underwent BCS and post-lumpectomy whole breast radiation treatment with a close margin did not have a higher rate of local recurrence compared to those with a wider margin width.^58^^,^^59^ Clasier et al.^40^ also concluded that a 2-mm margin may not be necessary if comprehensive surface imaging is achieved. Therefore, the clinical priority to intraoperatively detect close but negative margins for pure DCIS cases may decrease in the future.

Second, it was challenging to obtain accurate coregistration between DUV and corresponding H&E images. While DUV fluorescence images were taken from the top 20  μm of samples due to the shallow penetration depth of DUV light, routine H&E slide cutting techniques produces H&E images up to 200  μm into the FFPE tissue block. The optical sectioning thickness of ∼20  μm is also thicker than a typical paraffin-embedded thickness of ∼4  μm, which can cause subtle differences between DUV and H&E images. For instance, tubular-shape structures such as ducts and blood vessels usually have optically clear lumina in H&E images, but this is not the case in fluorescence images (Fig. 3). In Figs. 4(a) and 4(b), one focus of DCIS appears in the H&E image but is not easily identifiable in the fluorescence image. In Fig. 5(a), a topology of tissue compression and surface folding, which may be minimum is in whole lumpectomy specimens, is visible in the fluorescence image but was not observed in the H&E images.

Ideally, the segmentation algorithm accurately identifies all nuclei in the background, but this is difficult to achieve with simple edge detection and intensity thresholding because of the complicated textures and nuclei stacking. Fluorescence signals from cells slightly below the specimen surface can cause blurring and reduced intensity in nuclei images due to scattering, thus contributing to error in N/C calculation. A more advanced nuclei segmentation algorithm is needed for more accurate identification of cell nuclei in future studies. Some images showed a few small out of focus areas due to the irregular surface of fresh sliced tissue samples. Using the 4× objective lens plus tissue flattening significantly reduced blurring and thus minimized its impact on the accuracy of the device, particularly when visual evaluation was used. Acquiring image stacks for depth of field correction is an excellent way to obtain clear images.^46^ However, this approach may not be clinically practical because it takes at least five times longer for image acquisition and processing. The DUV-FSM device has the capability of autofocusing and we will investigate the autofocusing option in our future studies. Benign structures such as adenosis, blood vessels, and ducts may also include regions with higher N/C. Given the small size of the vessels and ducts, N/C of these tissue types can be averaged down using a large patch size. In this study, $2\times 2\;{\mathrm{mm}}^{2}$ patch size was selected for the calculation of N/C to be compatible with standard histopathology, which samplings at a step of 2 mm. An optimal image patch size may be determined by comparing the ROC curves obtained at different patches with more samples.

The 285-nm LED is capable of exciting PI and EY at the same time. PI stains cell nuclei, which is the primary contrast for detection of invasive samples in this study. EY stains connective tissue and cytoplasm. While EY staining is not very useful in the N/C method, it allows visualization of tissue structures beyond cell nuclei (otherwise would be dark), which is very useful in the visual evaluation method and potentially in a machine learning algorithm. We did notice the EY pooling effects and will explore ways to mitigate this effect in our future studies. Finally, the number of breast samples that have been imaged is relatively small. More samples are needed to calculate sensitivity and specificity for detection of invasive cancer more accurately. It also takes the current DUV-FSM about 1 min to image a ${1\text{-}\mathrm{cm}}^{2}$ breast specimen. Improvements in the motorized XY stage and scanning algorithm coupled with faster image transfer rate should reduce the scan time by a factor of at least 10.

## 5 Conclusions

In conclusion, a standard inverted microscope has been converted to a DUV-FSM as a potentially promising intraoperative tool for fast imaging of breast margins during BCS. Preliminary fluorescence images show excellent visual contrast in color, tissue texture, cell density, and shape between ILC, IDC, and normal breast tissues. Visual examination of MUSE images with minimal training achieved excellent accuracy in detecting invasive carcinoma from normal tissues. Statistical analysis identified significant differences in N/C between cancerous and normal tissue and achieved reasonably good sensitivity and specificity. While further work is needed to optimize DUV-FSM, future studies are also needed to explore the potential of DUV-FSM for intraoperative lumpectomy margin assessment.

## References

[1] Kummerow K. L. et al., “Nationwide trends in mastectomy for early-stage breast cancer,” JAMA Surg. 150(1), 9–16 (2015). 10.1001/jamasurg.2014.2895 25408966

[2] Kantor O. et al., “Impact of the Society of Surgical Oncology-American Society for Radiation Oncology Margin Guidelines on Breast—conserving surgery and mastectomy trends,” J. Am. Coll. Surg. 229(1), 104–114 (2019). JACSEX 1072-7515 10.1016/j.jamcollsurg.2019.02.051 30902638

[3] Marinovich M. L. et al., “The Association of Surgical Margins and Local Recurrence in women with ductal carcinoma in situ treated with breast-conserving therapy: a meta-analysis,” Ann. Surg. Oncol. 23(12), 3811–3821 (2016). 10.1245/s10434-016-5446-2 27527715 PMC5160992

[r4] Houssami N. et al., “The association of surgical margins and local recurrence in women with early-stage invasive breast cancer treated with breast-conserving therapy: a meta-analysis,” Ann. Surg. Oncol. 21(3), 717–730 (2014). 10.1245/s10434-014-3480-5 24473640 PMC5705035

[5] Donker M. et al., “Breast-conserving treatment with or without radiotherapy in ductal carcinoma in situ: 15-year recurrence rates and outcome after a recurrence, from the EORTC 10853 randomized phase III trial,” J. Clin. Oncol. 31(32), 4054–4059 (2013). JCONDN 0732-183X 10.1200/JCO.2013.49.5077 24043739

[6] Olsen M. A. et al., “Increased risk of surgical site infection among breast-conserving surgery re-excisions,” Ann. Surg. Oncol. 22(6), 2003–2009 (2015). 10.1245/s10434-014-4200-x 25358666 PMC4693603

[7] Greenup R. A. et al., “Cost implications of the SSO-ASTRO consensus guideline on margins for breast-conserving surgery with whole breast irradiation in stage I and II invasive breast cancer,” Ann. Surg. Oncol. 21(5), 1512–1514 (2014). 10.1245/s10434-014-3605-x 24577813

[8] Schulman A. M. et al., “Reexcision surgery for breast cancer: an analysis of the American Society of Breast Surgeons (ASBrS) Mastery(SM) database following the SSO-ASTRO ‘No Ink on Tumor’ guidelines,” Ann. Surg. Oncol. 24(1), 52–58 (2017). 10.1245/s10434-016-5516-5 27581607

[r9] Morrow M. et al., “Trends in reoperation after initial lumpectomy for breast cancer: addressing overtreatment in surgical management,” JAMA Oncol. 3(10), 1352–1357 (2017). 10.1001/jamaoncol.2017.0774 28586788 PMC5710510

[r10] Kaczmarski K. et al., “Surgeon re-excision rates after breast-conserving surgery: a measure of low-value care,” J. Am. Coll. Surg. 228(4), 504–512.e2 (2019). JACSEX 1072-7515 10.1016/j.jamcollsurg.2018.12.043 30703538

[r11] Havel L. et al., “Impact of the SSO-ASTRO margin guideline on rates of re-excision after lumpectomy for breast cancer: a meta-analysis,” Ann. Surg. Oncol. 26(5), 1238–1244 (2019). 10.1245/s10434-019-07247-5 30790112

[12] Schwarz J. Schmidt H., “Technology for intraoperative margin assessment in breast cancer,” Ann. Surg. Oncol. 27(7), 2278–2287 (2020). 10.1245/s10434-020-08483-w 32350717

[13] John E. R. S. et al., “Diagnostic accuracy of intraoperative techniques for margin assessment in breast cancer surgery: a meta-analysis,” Ann. Surg. 265(2), 300–310 (2017). 10.1097/SLA.0000000000001897 27429028

[14] Graham R. A. et al., “The efficacy of specimen radiography in evaluating the surgical margins of impalpable breast carcinoma,” AJR Am. J. Roentgenol. 162(1), 33–36 (1994). 10.2214/ajr.162.1.8273685 8273685

[15] Park K. U. et al., “Digital breast tomosynthesis for intraoperative margin assessment during breast-conserving surgery,” Ann. Surg. Oncol. 26(6), 1720–1728 (2019). 10.1245/s10434-019-07226-w 30877499

[16] Amer H. A. et al., “Digital breast tomosynthesis versus full-field digital mammography-Which modality provides more accurate prediction of margin status in specimen radiography?” Eur. J. Radiol. 93, 258–264 (2017). EJRADR 0720-048X 10.1016/j.ejrad.2017.05.041 28668424

[17] Olson T. P. et al., “Frozen section analysis for intraoperative margin assessment during breast-conserving surgery results in low rates of re-excision and local recurrence,” Ann. Surg. Oncol. 14(10), 2953–2960 (2007). 10.1245/s10434-007-9437-1 17674109

[18] Keating J. J. et al., “Advances in intraoperative margin assessment for breast cancer,” Curr. Surg. Rep. 4, 15 (2016). 10.1007/s40137-016-0136-3

[19] Gray R. J. et al., “Intraoperative margin management in breast-conserving surgery: a systematic review of the literature,” Ann. Surg. Oncol. 25(1), 18–27 (2018). 10.1245/s10434-016-5756-4 28058560

[20] Schnabel F. et al., “A randomized prospective study of lumpectomy margin assessment with use of MarginProbe in patients with nonpalpable breast malignancies,” Ann. Surg. Oncol. 21(5), 1589–1595 (2014). 10.1245/s10434-014-3602-0 24595800 PMC3975090

[21] LeeVan E. et al., “Use of MarginProbe as an adjunct to standard operating procedure does not significantly reduce re-excision rates in breast conserving surgery,” Breast Cancer Res. Treat. 183(1), 145–151 (2020). BCTRD6 10.1007/s10549-020-05773-5 32607640

[22] Zysk A. M. et al., “Intraoperative assessment of final margins with a handheld optical imaging probe during breast-conserving surgery may reduce the reoperation rate: results of a multicenter study,” Ann. Surg. Oncol. 22(10), 3356–3362 (2015). 10.1245/s10434-015-4665-2 26202553 PMC4839389

[r23] Zhou C. et al., “Integrated optical coherence tomography and microscopy for *ex vivo* multiscale evaluation of human breast tissues,” Cancer Res. 70(24), 10071–10079 (2010). CNREA8 0008-5472 10.1158/0008-5472.CAN-10-2968 21056988 PMC3028517

[r24] Nguyen F. T. et al., “Intraoperative evaluation of breast tumor margins with optical coherence tomography,” Cancer Res. 69(22), 8790–8796 (2009). CNREA8 0008-5472 10.1158/0008-5472.CAN-08-4340 19910294 PMC2782920

[25] Kennedy B. F. et al., “Investigation of optical coherence microelastography as a method to visualize cancers in human breast tissue,” Cancer Res. 75(16), 3236–3245 (2015). CNREA8 0008-5472 10.1158/0008-5472.CAN-14-3694 26122840

[26] Yu B. Ramanujam N., “Quantitative diffuse reflectance imaging of tumor margins,” in Biomedical Photonics Handbook, Vo-Dinh T., Ed., 2nd ed, pp. 565–586, CRC Press (2015).

[r27] Nichols B. S. et al., “A quantitative diffuse reflectance imaging (QDRI) system for comprehensive surveillance of the morphological landscape in breast tumor margins,” PLoS One 10(6), e0127525 (2015). POLNCL 1932-6203 10.1371/journal.pone.0127525 26076123 PMC4468201

[28] Brown J. Q. et al., “Optical assessment of tumor resection margins in the breast,” IEEE J. Sel. Top. Quantum Electron. 16, 530–544 (2010). IJSQEN 1077-260X 10.1109/JSTQE.2009.2033257 21544237 PMC3085495

[29] Keller M. D. et al., “Autofluorescence and diffuse reflectance spectroscopy and spectral imaging for breast surgical margin analysis,” Lasers Surg. Med. 42(1), 15–23 (2010). LSMEDI 0196-8092 10.1002/lsm.20865 20077490

[30] Keller M. D. et al., “Development of a spatially offset Raman spectroscopy probe for breast tumor surgical margin evaluation,” J. Biomed. Opt. 16(7), 077006 (2011). JBOPFO 1083-3668 10.1117/1.3600708 21806286 PMC3144975

[31] Xi L. et al., “Evaluation of breast tumor margins *in vivo* with intraoperative photoacoustic imaging,” Opt. Express 20(8), 8726–8731 (2012). OPEXFF 1094-4087 10.1364/OE.20.008726 22513583

[r32] Wong T. T. W. et al., “Fast label-free multilayered histology-like imaging of human breast cancer by photoacoustic microscopy,” Sci. Adv. 3(5), e1602168 (2017). STAMCV 1468-6996 10.1126/sciadv.1602168 28560329 PMC5435415

[33] Li R. et al., “Assessing breast tumor margin by multispectral photoacoustic tomography,” Biomed. Opt. Express 6(4), 1273–1281 (2015). BOEICL 2156-7085 10.1364/BOE.6.001273 25909011 PMC4399666

[34] Tummers Q. R. et al., “Real-time intraoperative detection of breast cancer using near-infrared fluorescence imaging and methylene blue,” Eur. J. Surg. Oncol. 40(7), 850–858 (2014). 10.1016/j.ejso.2014.02.225 24862545 PMC4035701

[35] Patel R. et al., “Multimodal optical imaging for detecting breast cancer,” J. Biomed. Opt. 17(6), 066008 (2012). JBOPFO 1083-3668 10.1117/1.JBO.17.6.066008 22734764

[36] Mazhar A. et al., “Wavelength optimization for rapid chromophore mapping using spatial frequency domain imaging,” J. Biomed. Opt. 15(6), 061716 (2010). JBOPFO 1083-3668 10.1117/1.3523373 21198164 PMC3031903

[37] Laughney A. M. et al., “Spectral discrimination of breast pathologies in situ using spatial frequency domain imaging,” Breast Cancer Res. 15(4), R61 (2013). BCTRD6 10.1186/bcr3455 23915805 PMC3979079

[38] Dixon J. M. et al., “Intra-operative assessment of excised breast tumour margins using ClearEdge imaging device,” Eur. J. Surg. Oncol. 42(12), 1834–1840 (2016). 10.1016/j.ejso.2016.07.141 27591938

[39] St John E. et al., “Real time intraoperative classification of breast tissue with the intelligent knife,” Eur. J. Surg. Oncol. 42(5), S25 (2016). 10.1016/j.ejso.2016.02.102

[40] Glaser A. K. et al., “Light-sheet microscopy for slide-free non-destructive pathology of large clinical specimens,” Nat. Biomed. Eng. 1(7), 0084 (2017). 10.1038/s41551-017-0084 29750130 PMC5940348

[41] Chen Y. et al., “Rapid pathology of lumpectomy margins with open-top light-sheet (OTLS) microscopy,” Biomed. Opt. Express 10(3), 1257–1272 (2019). BOEICL 2156-7085 10.1364/BOE.10.001257 30891344 PMC6420271

[42] Maloney B. W. et al., “Review of methods for intraoperative margin detection for breast conserving surgery,” J. Biomed. Opt. 23(10), 100901 (2018). JBOPFO 1083-3668 10.1117/1.JBO.23.10.100901 PMC6210801 30369108

[43] Dumitru D. Douek M. Benson J. R., “Novel techniques for intraoperative assessment of margin involvement,” Ecancermedicalscience 12, 795 (2018). 10.3332/ecancer.2018.795 29434661 PMC5804713

[44] Fereidouni F. et al., “Microscopy with ultraviolet surface excitation for rapid slide-free histology,” Nat. Biomed. Eng. 1(12), 957–966 (2017). 10.1038/s41551-017-0165-y PMC6223324 31015706

[r45] Yoshitake T. et al., “Rapid histopathological imaging of skin and breast cancer surgical specimens using immersion microscopy with ultraviolet surface excitation,” Sci. Rep. 8(1), 4476 (2018). SRCEC3 2045-2322 10.1038/s41598-018-22264-2 29540700 PMC5852098

[46] Xie W. et al., “Microscopy with ultraviolet surface excitation for wide-area pathology of breast surgical margins,” J. Biomed. Opt. 24(2), 026501 (2019). JBOPFO 1083-3668 10.1117/1.JBO.24.2.026501 PMC6368047 30737911

[47] Moran M. S. et al., “Society of Surgical Oncology—American Society for Radiation Oncology consensus guideline on margins for breast-conserving surgery with whole-breast irradiation in stages I and II invasive breast cancer,” J. Clin. Oncol. 32(14), 1507–1515 (2014). JCONDN 0732-183X 10.1200/JCO.2013.53.3935 24516019

[48] Schindelin J. et al., “Fiji: an open-source platform for biological-image analysis,” Nat. Methods 9, 676–682 (2012). 10.1038/nmeth.2019 22743772 PMC3855844

[49] Peng T. et al., “A BaSiC tool for background and shading correction of optical microscopy images,” Nat. Commun. 8, 14836 (2017). NCAOBW 2041-1723 10.1038/ncomms14836 28594001 PMC5472168

[50] Preibisch S. Saalfeld S. Tomancak P., “Globally optimal stitching of tiled 3D microscopic image acquisitions,” Bioinformatics 25(11), 1463–1465 (2009). BOINFP 1367-4803 10.1093/bioinformatics/btp184 19346324 PMC2682522

[51] Wu X. et al., “Label-free detection of breast masses using multiphoton microscopy,” PLoS One 8(6), e65933 (2013). POLNCL 1932-6203 10.1371/journal.pone.0065933 23755295 PMC3675049

[52] Saad R. S. Silverman J. F., “Breast,” Chapter 25 in Comprehensive Cytopathology, Bibbo M. Wilbur D., Eds., 3rd ed, pp. 713–772, W.B. Saunders, Edinburgh (2008).

[53] Gonzalez R. C. Richard E., Woods, Digital Image Processing, Vol. 8, pp. 567–612, Prentice Hall Press (2002).

[54] Liang K.-Y. Zeger S. L., “Longitudinal data analysis using generalized linear models,” Biometrika 73(1), 13–22 (1986). BIOKAX 0006-3444 10.1093/biomet/73.1.13

[55] Kotwall C. et al., “Relationship between initial margin status for invasive breast cancer and residual carcinoma after re-excision,” Am. Surg. 73(4), 337–343 (2007). AJOOA7 0096-6347 10.1177/000313480707300405 17439024

[56] Childers C. P. Maggard-Gibbons M., “Understanding costs of care in the operating room,” JAMA Surg. 153(4), e176233 (2018). 10.1001/jamasurg.2017.6233 29490366 PMC5875376

[57] Morrow M. et al., “Society of Surgical Oncology-American Society for Radiation Oncology-American Society of Clinical Oncology consensus guideline on margins for breast-conserving surgery with whole-breast irradiation in ductal carcinoma in situ,” Ann. Surg. Oncol. 23(12), 3801–3810 (2016). 10.1245/s10434-016-5449-z 27527714 PMC5047939

[58] Van Zee K. J. et al., “Relationship between margin width and recurrence of ductal carcinoma *in situ*: analysis of 2996 women treated with breast-conserving surgery for 30 years,” Ann. Surg. 262(4), 623–631 (2015). 10.1097/SLA.0000000000001454 26366541 PMC4739638

[59] Tadros A. B. et al., “Ductal carcinoma *in situ* and margins <2 mm: contemporary outcomes with breast conservation,” Ann. Surg. 269(1), 150–157 (2019). 10.1097/SLA.0000000000002439 28742682 PMC6051916

